# Supplementary material for: Fabrication and Evaluation of Alginate/Bacterial Cellulose Nanocrystals–Chitosan–Gelatin Composite Scaffolds
Source: Molecules. 2021 Aug 18;26(16):5003. doi: 10.3390/molecules26165003 (PMC8400783; doi:10.3390/molecules26165003)
Supplement: Supplementary file 1 [file molecules-26-05003-s001.zip › molecules-1291734-supplementary.pdf]

molecules

Supplementary Materials

Fabrication and Evaluation of Alginate/Bacterial Cellulose Nanocrystals–Chitosan–Gelatin Composite Scaffolds

Zhengyue Li [1,2,3], Xiuqiong Chen [1,2,3], Chaoling Bao [2,3], Chang Liu [2,3], Chunyang Liu [2,3], Dongze Li [2,3], Huiqiong Yan [1,2,3,]* and Qiang Lin [1,2,3,]*

[1] Key Laboratory of Tropical Medicinal Resource Chemistry of Ministry of Education, College of Chemistry and Chemical Engineering, Hainan Normal University, Haikou 571158, China; zhengyedu@163.com (Z.L.); chenxiuqiongedu@163.com (X.C.)
[2] Key Laboratory of Natural Polymer Functional Material of Haikou City, College of Chemistry and Chemical Engineering, Hainan Normal University, Haikou 571158, China; bcl0409@163.com (C.B.); liuchang202107@126.com (C.L.); liuchunyang212021@126.com (C.L.); dongzeli2019@163.com (D.L.)
[3] Key Laboratory of Water Pollution Treatment & Resource Reuse of Hainan Province, College of Chemistry and Chemical Engineering, Hainan Normal University, Haikou 571158, China
* Correspondence: yanhqedu@163.com (H.Y.); linqiang@hainu.edu.cn (Q.L.); Tel.: +86-0898-6588-4995 (H.Y.); +86-0898-6588-9422 (Q.L.)

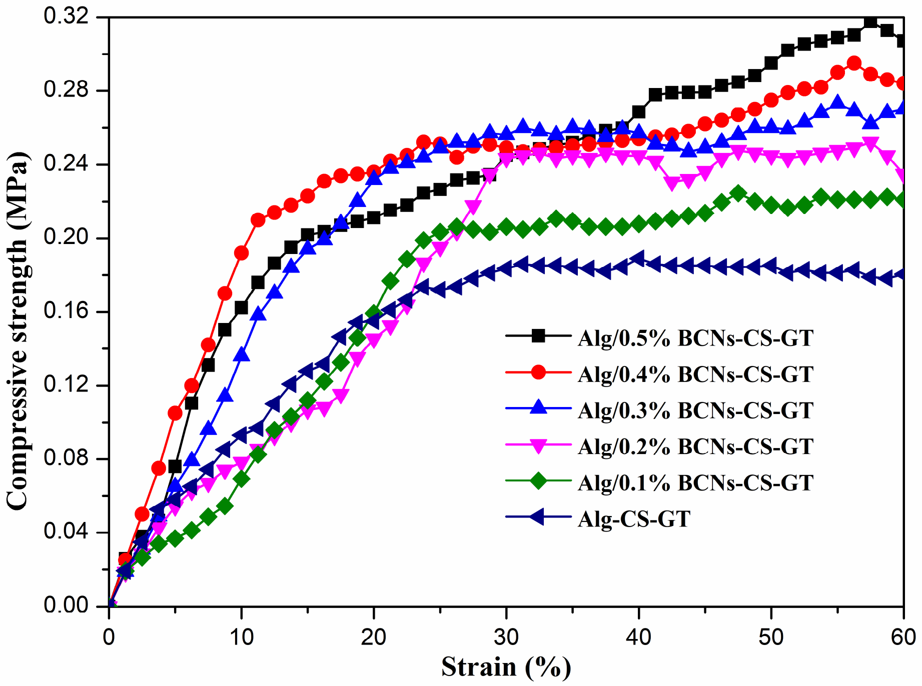

Figure S1. Stress-strain curves of the composite scaffolds.

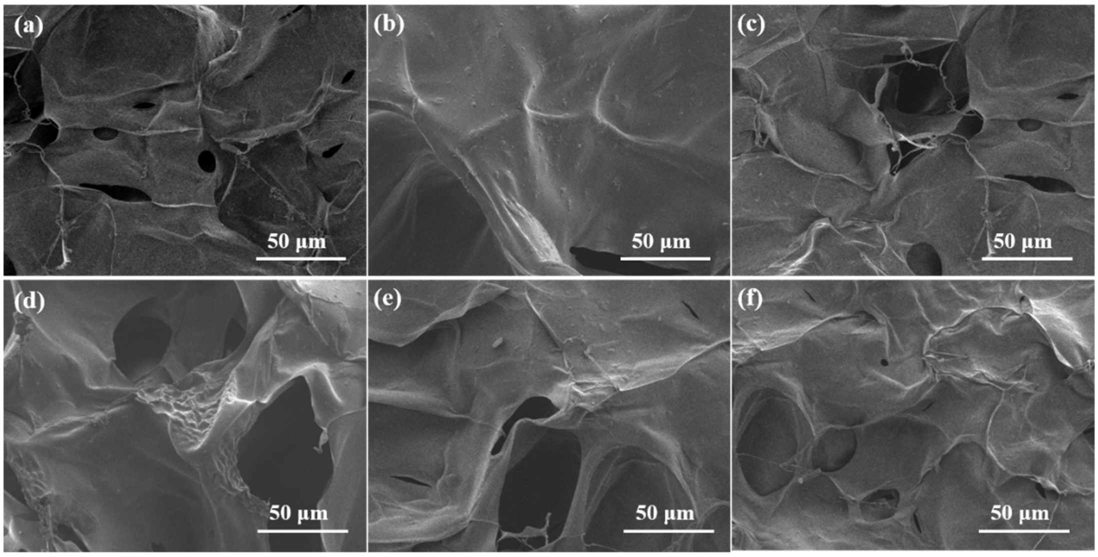

Figure S2. SEM images of the composite scaffolds without cells after incubation for 2 days: (**a**) Alg-CS-GT, (**b**) Alg/0.1% BCNs-CS-GT, (**c**) Alg/0.2% BCNs-CS-GT, (**d**) Alg/0.3% BCNs-CS-GT, (**e**) Alg/0.4% BCNs-CS-GT and (**f**) Alg/0.5% BCNs-CS-GT.

Table S1. Ca/P ratios evaluated based on EDX analysis after 14 days of incubation in SBF.

Sample	Ca/P ratio	Crystalline structure
Alg-CS-GT composite scaffolds	1.52	Apatite
Alg/0.3% BCNs-CS-GT composite scaffolds	1.59	Apatite
